# Break the Spasm with Succinylcholine, but Risk Intraoperative Awareness with Undiagnosed Pseudocholinesterase Deficiency

**DOI:** 10.1155/2020/8874617

**Published:** 2020-09-18

**Authors:** Danny D. Bui, Shyamal R. Asher

**Affiliations:** Rhode Island Hospital, Department of Anesthesiology, The Warren Alpert Medical School of Brown University, 593 Eddy St Davol 129, Providence, RI 02903, USA

## Abstract

Succinylcholine is a commonly used medication in all aspects of anesthetic care, and there are a number of known side effects and complications associated with its use. However, when succinylcholine is used emergently, anesthesia providers must remain vigilant to undiagnosed conditions that pose additional risks to patients. We report the use of succinylcholine to treat acute, refractory laryngospasm after extubation leading to prolonged neuromuscular paralysis. There are unique challenges presented by this case including the risk of anesthesia awareness with recall due to the cognitive biases that prevent the clinical diagnosis of pseudocholinesterase deficiency.

## 1. Introduction

Succinylcholine, a depolarizing muscle relaxant, is a critical medication that can be used in all aspects of anesthetic care. At induction, succinylcholine is used to quickly optimize intubating conditions due to its rapid onset and typically short duration of action. Intraoperatively, succinylcholine can be used in procedures that require brief periods of intense paralysis such as ear, nose, and throat (ENT) procedures. At emergence, succinylcholine is a vital medication in emergencies such as refractory laryngospasm after extubation. There are a number of known side effects and potential complications of succinylcholine. However, these may not be recognized during an emergency, thus placing the patient at additional risk of harm. We present such a case where succinylcholine was emergently and successfully used to treat acute, refractory laryngospasm following extubation, but this was followed by prolonged neuromuscular blockade. As discussed elsewhere [1, 2], pseudocholinesterase deficiency is a well-known condition that can significantly prolong succinylcholine activity, but this condition can be undiagnosed at the time of presentation. When succinylcholine is needed emergently at the time of emergence, it can place patients with undiagnosed pseudocholinesterase deficiency at increased risk of airway complications and anesthesia awareness with recall.

## 2. Case Description

A 30-year-old female with a history of type-1 diabetes and cholelithiasis presented with five days of intermittent right upper quadrant pain, nausea, and emesis. Elective laparoscopic cholecystectomy was scheduled. The patient had an American Society of Anesthesiology (ASA) physical status classification of 2, weighed 69.9 kg with a body mass index (BMI) of 24.8, and had no prior surgeries or family history of anesthesia-related complications. The anesthetic plan included general anesthesia with an endotracheal tube, and induction medications included 200 mg of propofol (∼3 mg/kg) and 50 mg of rocuronium (0.7 mg/kg). The patient was hemodynamically stable during the surgery that had an operative time of 40 minutes. Redosing of rocuronium was not required, and the patient was reversed with 200 mg of sugammadex with a train of four (TOF) monitoring revealing 4/4 twitches prior to emergence.

After extubation, poor air movement was noted leading to a suspected clinical diagnosis of laryngospasm that did not improve with jaw thrust maneuvers and positive pressure. With a declining oxygen saturation, 40 mg (∼0.5 mg/kg) of intravenous (IV) succinylcholine was emergently administered with rapid resolution of laryngospasm. The oxygen saturation nadir was 79% but rapidly improved with easy mask ventilation. Thereafter, the emergence process was continued. However, the patient did not recover spontaneous ventilation immediately. After approximately 11 minutes, the patient was noted to become hypertensive to systolic of 175 mmHg and tachycardic to 127 beats per minute. A diagnosis of pseudocholinesterase deficiency was suspected, and the patient was sedated again with 2 mg of IV midazolam and 100 mg of IV propofol and reintubated successfully. Repeated TOF monitoring revealed 0/4 twitches.

The patient was brought to the postanesthesia care unit (PACU) with continued mechanical ventilation and sedation with a propofol infusion at 100–150 mcg/kg/min where she remained hemodynamically stable. TOF monitor was periodically checked in the PACU. Approximately 2.5 hours later, TOF monitor revealed 4/4 twitches with no fade and the patient was successfully weaned of mechanical ventilation and extubated with no further complications. The patient noted mild chest tightness and right-sided pleuritic chest pain. However, there was no recall of perioperative events.

The patients' blood samples were sent to the laboratory for a cholinesterase and dibucaine test. Since these tests are sent outside the hospital laboratory, the results were obtained three days later and confirmed the suspected clinical diagnosis of pseudocholinesterase deficiency (Table 1).

## 3. Discussion

Succinylcholine is an important medication in the armamentarium of anesthesia providers with its use spanning the entire spectrum of anesthetic care. There are a number of known side effects and complications associated with succinylcholine use that can be planned for and avoided when known preoperatively. However, when succinylcholine is used emergently, anesthesia providers must remain vigilant to undiagnosed conditions that pose additional risks to patients.

We report one such situation with undiagnosed pseudocholinesterase deficiency. Succinylcholine is normally metabolized by enzymes synthesized by the liver known as pseudocholinesterase. As discussed by Andersson [1], pseudocholinesterase deficiency can result from congenital defects or acquired events such as liver failure and drug interactions. The use of succinylcholine in the setting of genetic pseudocholinesterase deficiency leads to prolonged paralysis that can last for several hours. As discussed by Trujillo [2], these patients are treated with supportive measures including mechanical ventilation and sedation until the succinylcholine is naturally excreted.

There are a number of case reports elsewhere [3–5] on succinylcholine use at induction leading to prolonged paralysis and need for prolonged mechanical ventilation due to undiagnosed pseudocholinesterase deficiency. However, the use of succinylcholine at emergence in the setting of undiagnosed pseudocholinesterase deficiency poses additional unique challenges and risks to the patient including challenging airway management and risk of anesthesia awareness with recall.

First, airway management may be more challenging after emergence than at induction. The risk factors for a more challenging airway at the end of the surgery include prolonged duration of surgery, Trendelenburg position, and the need for large volume resuscitation. In our case, the patient was easy to mask ventilate and intubate at the end of surgery with the short and straightforward nature of the surgery.

In addition, succinylcholine use at emergence may place the patient at increased risk of anesthesia awareness in the setting of undiagnosed pseudocholinesterase deficiency. As reported by Cascella [6], anesthesia awareness is a rare complication with current estimates of incidence ranging from 0.1 to 0.2%. However, in a study by Thomsen [7] specifically assessing patients with pseudocholinesterase deficiency, up to 50% reported awareness under anesthesia at the time of emergence leading to severe psychological trauma. In all these cases, succinylcholine was used at induction, and the lack of intraoperative TOF monitoring was determined to be the major risk factor for awareness at emergence. In our case, TOF monitoring was performed intraoperatively, and the small dose of succinylcholine may not have caused complete paralysis in a patient with normal pseudocholinesterase activity. Therefore, the emergence process was continued while supporting ventilation placing the patient, with undiagnosed pseudocholinesterase deficiency, at significant risk of anesthesia awareness with recall. Fortunately, the changes in vital signs were quickly recognized and treated with amnestic and hypnotic agents which likely prevented awareness, while the diagnosis and management of the patient continued.

This case also brings to light the cognitive processes involved in decision-making within anesthesiology practice. Anesthesia care requires making rapid and complex decisions with limited data that is susceptible to decision-making errors. We commonly use cognitive short cuts, known as heuristics by Stiegler [8], to make decisions quickly and efficiently, but these can sometimes lead to an incorrect decision. Heuristics such as anchoring, where one fails to modify the initial diagnosis due to new events and confirmation bias, where one only seeks information that supports their diagnosis, could have led to an adverse outcome in this patient. For example, prior to emergence, the patient's neuromuscular blockade was reversed and TOF monitoring revealed 4/4 twitches. After the laryngospasm event had resolved, anchoring to the prior 4/4 twitches would have resulted in failure to recognize residual neuromuscular paralysis in the patient raising the risk of awareness. As discussed elsewhere [8, 9], it is important to recognize the nonrational cognitive factors involved in decision-making in order to develop self-awareness and counterbalance our intuitive decision-making.

This case report illustrates how undiagnosed pseudocholinesterase deficiency can place a patient at increased risk of anesthesia awareness when succinylcholine use is necessary at the time of emergence. When the emergence process is continued, anesthesia providers must remain vigilant and recognize the limitations of cognitive heuristics involved in the decision-making process in order to avoid devastating outcomes such as anesthesia awareness with recall (Table 2).

## Figures and Tables

**Table 1:** Results of the patient's serum cholinesterase level and the percent inhibition by dibucaine.

	Reference range	Patient result
Cholinesterase, plasma	2673–6592 IU/L	608 (L)
Dibucaine inhibition	81.6–88.3% inhibition	34.8 (L)

Dibucaine inhibition is the percent of the pseudocholinesterase enzyme inhibited by the local anesthetic dibucaine. A dibucaine number less than 30 in addition to decreased plasma cholinesterase activity indicates a patient at high risk for prolonged paralysis after succinylcholine use [1].

**Table 2:** Timeline.

Time	Event
09: 35	IV induction with 200 mg propofol, 50 mg rocuronium, and 100 mcg fentanyl
09: 39	Intubation
09: 54	Procedure start
10: 34	Procedure end, 4/4 twitches on TOF monitoring after reversal with sugammadex
10: 35	Emergence
10: 40	Extubation; laryngospasm; 40 mg IV succinylcholine administered
10: 50	No spontaneous respiratory effort; blood pressure increased from 100/57 to 175/22 and heart rate from 64 to 127; 0/4 twitches on recheck of TOF
10: 51	Midazolam 2 mg and propofol 100 mg IV administered; the patient was reintubated and started on a propofol drip
11: 33	Transport to PACU intubated and sedated
11: 40	Labs for dibucaine number and plasma cholinesterase sent
13: 30	4/4 twitches on TOF monitor, sedation weaned, patient extubated once awake and following commands
16: 05	Patient reports well-controlled pain and no awareness of perioperative events
